# Isobolographic analysis reveals antinociceptive synergism between Phα1β recombinant toxin and morphine in a model of cancer pain in C57BL/6J mice

**DOI:** 10.1590/1678-9199-JVATITD-2021-0027

**Published:** 2021-08-25

**Authors:** Caio Tavares Aoki, Rodrigo Andrade Moura, Luana Assis Ferreira, Mariana Garcia Mendes, Duana Carvalho Santos, Marcio Junior Rezende, Marcus Vinícius Gomez, Célio José Castro-Junior

**Affiliations:** 1 Graduate Program in Health Sciences, Institute of Education and Research, Santa Casa de Belo Horizonte, Belo Horizonte, MG, Brazil.

**Keywords:** Cancer pain, Melanoma, Morphine, Phα1β, Synergism, Isobolographic analysis

## Abstract

**Background:**

**Phoneutria nigriventer** venom contains Phα1β. This toxin and its recombinant form have a remarkable analgesic potential that is associated with blockage of voltage-gated calcium channels and TRPA1 receptors. Although morphine is a mainstay drug to treat moderate and severe pain related to cancer, it has serious and dose-limiting side effects. Combining recombinant Phα1β and morphine to treat pain is an interesting approach that has been gaining attention. Therefore, a quantitative and reliable method to establish the strength of the antinociceptive interaction between these two substances is necessary. The present study was designed to investigate the nature of the functional antinociceptive (analgesic) interaction between Phα1β recombinant toxin and morphine in a model of cancer pain.

**Methods:**

Melanoma was produced by intraplantar inoculation of B16-F10 cells into the right paw of C57BL/6J mice. Von Frey filaments measured the paw-withdrawal threshold after intrathecal administration of morphine, recombinant Phα1β, and their combination. Thermal hyperalgesia was assessed using Hargreaves apparatus. The degree of interaction was evaluated using isobolographic analysis. Spontaneous and forced motor performance was assessed with the open-field and rotarod tests, respectively.

**Results:**

Co-administration of recombinant Phα1β and morphine synergistically reverses the melanoma-induced mechanical hyperalgesia. The potency of the mixture, measured as the effective dose to reach 50% of maximum possible effect (MPE) in ameliorating mechanical hyperalgesia, was about twice fold higher than expected if the interaction between morphine and recombinant Phα1β was merely additive. Treatment with the combination at doses necessary to reach 50% of MPE caused no spontaneous nor forced motor alterations.

**Conclusion:**

The combinatorial use of recombinant Phα1β and morphine allows significant and effective dose reduction of both agents, which has translational potential for opioid-sparing approaches in pain management related to cancer.

## Background

**Figure 1.**
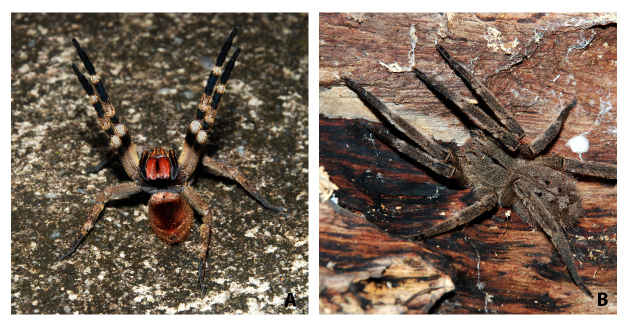
*Phoneutria nigriventer* spider. **(A)** Frontal view in its defensive position and **(B)** back view of adult species found in the central region of Minas Gerais state, Brazil. Photo by Pedro H. Martins, personal archives (reproduced with permission).

The Phα1β toxin, purified from the venom of the spider *Phoneutria nigriventer* (Figure 1), and its recombinant form have marked analgesic action demonstrated in both acute and chronic preclinical pain models [1]. This toxin has 55 amino acids on its sequence and is a dual blocker of voltage-sensitive calcium channels [2] and TRPA1 receptors [3]. This unique profile helps to explain its higher analgesic potency and efficacy compared to the antinociceptive effect of other calcium channel blocking analgesic toxins such as ω-conotoxin MVIIA [4]. Despite the growing number of evidence regarding the analgesic potential of Phα1β toxin, the majority of the studies so far present data in which the toxin is used alone.

The incidence and morbidity of cancer is a growing problem worldwide. The recent improvement of treatment options and diagnostic tools have been decreasing the lethality rate of cancer. Hence, the number of cancer survivors has been increasing. As a consequence, the number of people living with long-term symptoms or symptoms associated with cancer treatment is growing too. Pain is a prevalent symptom in cancer patients (30-65%) and has a severe impact on the quality of life of these individuals. In patients with advanced cancer, pain is described as moderate to severe in approximately 40 to 50% and very intense or excruciating in 25 to 30% of cases [5]. Therefore, better options for managing pain in cancer patients must be pursued.

Opioids represent the front line for the treatment of moderate and severe cancer-associated pain. Amongst opioid drugs, morphine is the cornerstone to be used in this scenario. Despite the good analgesic efficacy in acute treatment, continuous and long-term treatment with morphine induces tolerance, which requires successive dose increment [6]. This generates adverse effects such as constipation, sedation, itching, among others, which limits its use. Thus, the efficacy and safety of opioids require improvement for better pain management.

Combinatorial analgesic regimens are frequently used for the management of cancer pain particularly for patients who become unresponsive to the conventional therapy [7]. The most well studied combinations of opioids and other drugs are opioid-NSAID and opioid-α2 adrenergic agonist [8]. Evidence for the efficacy of the association between opioid and voltage-gated calcium channel blockers (VGCC) are scarce. There are few clinical and preclinical studies providing some support for the use of ziconotide in combination with morphine albeit strong evidence-based data are still limited [9]. 

Although acting on distinct targets, both morphine and the Phα1β toxin can reduce excitability in excitatory synapses in the dorsal horn of the spinal cord. The intracellular calcium concentration is an overlapping point downstream to µ-opioid receptor activation and VGCC blockage that could be subject to a cooperative interaction of the two drugs if they are used together. Recent studies suggest a favorable analgesic interaction between morphine and Phα1β when used together. The co-administration of Phα1β potentiated the antinociceptive action of morphine in the acute thermal pain model [10] and the post-surgical incisional pain model [11]. In another model of cancer-associated pain Rigo et al. [12] showed that the adjuvant use of Phα1β was able to reverse the analgesic tolerance induced by repeated administrations of morphine.

Thus, both mechanistic and preclinical evidence point toward a cooperative interaction between morphine and Phα1β to cause analgesia. However, a reliable and quantitative degree of the interaction between these two drugs is still unknown. Our study aimed to determine the degree of analgesic interaction, throughout isobolographic analysis, of the joint administration of Phα1β recombinant toxin and morphine in mice submitted to a model of chronic pain associated with melanoma and to establish whether the interaction is subadditive, additive, or synergistic as well as to determine whether such interaction occurs for adverse motor effects.

## Methods

### Animals

All the procedures were approved by the Ethics Committee in experimentation with living animals from Santa Casa Hospital of Belo Horizonte (Protocol 002/2018. Procedures complied with the guidelines of the International Association for the Study of Pain [13] and are in accordance with the ARRIVE guidelines for reporting experiments involving animals, Male C57BL/6J mice (20-30 g; 5-7 weeks old) were bred in house at a controlled temperature (22±2 ºC) under a 12-h light/dark cycle with standard laboratory chow and tap water available *ad libitum*. The animals were habituated to the experimental room 1 hour before each trial, 6 to 8 animals were used in each experimental group. 

### Drugs

Morphine sulphate pentahydrate injectable solution (Dimorf^®^ - Cristalia, Brazil, #17129456) was diluted in PBS to the following doses before its use (0.1 µg/site, 0.4 µg/site and 1.6 µg/site). The recombinant form of Phα1β was purchased from Giotto Biotech (Florence, Italy). The stock recombinant Phα1β (lyophilized) was dissolved in PBS to reach the following doses: 10 pmol/site, 30 pmol/site and 100 pmol/site. 

### Intrathecal injections

Intrathecal injections for morphine and recombinant Phα1β (i.t.) were performed following the method previously described [14]. Briefly, a volume of 5 μL was administered between the L5 and L6 lumbar vertebrae using a 10-μL Hamilton micro-syringe while the animal was restrained to maintain the position of the needle. Puncture of the dura was indicated behaviorally by a slight flick of the tail. Experimenters were blinded to the group allocation and drug treatment when performing tests.

### Tumor inoculation with B16-F10 cells

Melanoma cells from murine (B16-F10 - ATCC: CRL-6475; melanoma cell line) were incubated as monolayer cultures in Dulbecco’s modified Eagle’s medium (DMEM) containing 10% fetal bovine serum and 1% penicillin/streptomycin at 37^o^C with 5% CO2 in a humidified atmosphere. About 20 µL of melanoma cells (1 x 10^5^ cells) suspended in PBS were injected subcutaneously into the plantar region of the right hind paw of C57BL/6 mice [15].

### Thermal hyperalgesia assessment

The paw reaction test to a heat stimulus was performed as described previously [16]. A radiant light beam from a 60-W light bulb was directed onto the right hind paw (70% of the total intensity was used). The time between the onset of the stimulus and paw withdrawal was measured and used as an index of the thermal nociceptive threshold. A maximum latency of 30 s was imposed to prevent tissue damage. Data following drug treatments were further normalized by the baseline latency determined before B16-F10 inoculation.

### Mechanical threshold measurement by Von Frey filaments

Mice were acclimatized forty minutes before tests in individual, clear, Plexiglas boxes (9 × 7 × 11 cm) on an elevated, wire mesh platform to allow access to the plantar surface of both hind paws. The measurement of the mechanical threshold was carried out using the up-and-down method [17,18]. The von Frey filaments with increasing stiffness (0.01-4g) were presented to the right and left hind paws with pressure causing the filament to blend. The 1 g filament is the first to be presented, if the animal withdraws its paw a smaller filament is presented to it, if the animal does not remove the paw a stiffer filament is presented, the cutoff for longer filament is 4 g. At least six responses around the estimated threshold are required for optimal calculation of the 50% paw withdraw threshold (PWT) in grams. Measurements were taken before B16-F10 inoculation (baseline), on the seventh-day, on the fourteenth-day post-inoculation, and a post-drug treatment measurement. 

### Behavioral analysis on open field and rotarod test

We evaluated spontaneous and forced locomotor activity of animals that received the treatments using the open field and the rotarod tests. The open field apparatus consisted of a box measuring 25 x 25 cm with a floor divided into nine identical areas. Twenty-five minutes after injection of the tested drugs, the animal is placed in the apparatus allowing uninterrupted free movement of the mice in question along their maze quadrant for a single period of 300 seconds, during which the tracking software record all movement performed by the mice. Three parameters were evaluated for 300 seconds during the test: total movement distance (traveled distance), the number of rearing movements, and duration of these movements. To evaluation of forced locomotor activity, the rotarod test was used. Mice were exposed to a moving cylinder with constant acceleration and latency to the first fall was registered. Before being submitted to the rotarod test, the mice were trained in the cylinder for three consecutive days, as early described. Both open field and rotarod tests were performed with naive animals and not in mice with melanoma in order to overcome bias related to mechanical deficits associated to melanoma induction in the paw. 

## Data analysis

The results were expressed as mean ± S.E.M., ED_50_ values (amount of drug that produces half of the maximum response regarding the effectiveness of this drug) accompanied by their respective 95% confidence limits. Data were analyzed by one or two-way analysis of variance (ANOVA) followed by a post-test where appropriate. To determine the antinociceptive interaction between Phα1β and morphine we performed isobolographic analysis [19].

The reversal of melanoma-induced mechanical hyperalgesia was normalized to the percentage of the maximum possible effect (% MPE) according to the formula:

$$\%MPE=100\;\times \;\left(\frac{A-B}{C-B}\right)$$

wherein A is the PWT of each animal in the treated group (drugs alone or in combination). B is the PWT of the animal with melanoma-associated hyperalgesia and before treatment. C is the cutoff of the PWT herein set as 4g. To construct isobologram, dose-response curves were first obtained for morphine and Phα1β administered alone. Line equations, slope values ED_50_ values and 95% confidence limits were obtained using linear regression [19-21]. The slope values were used to assess whether the dose-effect of these drugs alone exhibited a constant potency ratio, which is necessary to perform a fixed dose-pair combination of drugs [19-21]. Doses of individual drugs in the combinations were determined as a proportion of their ED_50_ values. This proportion was constant and estimated based on a factor derived from the individual variances of the ED_50_ values. This fixed proportion of agents was necessary to assess whether the combination displayed enhanced potency indicative of synergism and was determined as it follows. The total amount of Phα1β+morphine in a drug pair was defined as:

a+b=c

wherein: a = the quantity (in pmol) of Phα1β; b = the quantity (in pmol) of morphine; c = sum (in pmol) of the quantities of Phα1β and morphine in the mixture. The proportion of a and b was fixed and calculated according to the formulae bellow:

$$\begin{array}{c} a=A\;\times \;\;f\\ b=\left(1-f\right)\times B \end{array}$$

wherein: A = ED_50_ of Phα1β; B = ED_50_ of morphine; f = proportion factor.

The proportion factor f was calculated based on the variances of the ED_50_ values from Phα1β (A) and morphine (B) according to the formula:

$$f=\;\frac{V\;(B)}{V\;\left(A\right)+V\;(B)}$$

wherein: V (A) = variance of ED_50_ of Phα1β and V (B) = variance of ED_50_ of morphine. 

Dose-response curves of associated drugs were constructed to obtain the doses that achieved the same effect level (50% MPE) compared to drugs given alone. This experimentally obtained ED_50_ (here called Z_mix_) was compared (*t-*test) to a theoretically calculated ED_50_ value for additive interactions (Z_add_). The Z_add_ was obtained according to the formula:

$$Z_{add}=f\;\times A+\left(1-f\right)\times B$$

wherein A = ED_50_ of Phα1β; B = ED_50_ of morphine; f = proportion factor. 

The variance of Z_add_ was estimated by the formula:

$$V(Z_{add})=\;f^{2}\;\times V\left(A\right)+(1-f\;{)}^{2}\;\times V\left(B\right)$$

wherein f = proportion factor; V(A) and V(B) = Variances of the ED_50_ of Phα1β and morphine, respectively. Graphical assessment of synergy were also presented using isobologram. Measurement of the interaction index (α) was obtained by dividing experimentally obtained ED_50_ of the drug pair by the theoretical additive ED_50_ of the drug pair. The γ interaction index provides a measure of the degree of synergism. The level of significance for all tests was set at p <0.05. GraphPad 7 was used for graph creation and statistical analysis. A custom-made spreadsheet was constructed by the authors in Excel software for isobolographic analysis.

## Results

### Melanoma cell injection induces mechanical and thermal hyperalgesia

**Figure 2.**
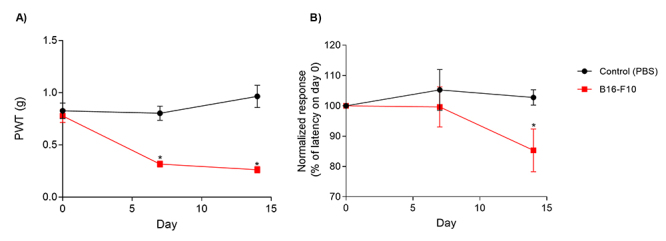
Melanoma cell injection induces mechanical and thermal hyperalgesia. **(A)** The development of mechanical hyperalgesia. There is a significant reduction in the paw withdraw threshold (PWT) at 7 and 14 days after B16-F10 cell inoculation. **(B)** Thermal hyperalgesia was seen 14 days after the B16-F10 injection. Both tests have assessed the right hind paw response. *p < 0.05 in comparison with day 0 (ANOVA with repeated measures). Data are expressed as mean ± SEM (n = 12 animals per group).

Intraplantar inoculation of melanoma cells, but not the vehicle (PBS), in C57BL/6 mice induced mechanical hyperalgesia at day 7 and become more pronounced at day 14 (Figure 2A). The paw withdrawal threshold (PWT) drops from 0.778 ± 0.063 g before melanoma inoculation to 0.262 ± 0.032 g at day 14 (p < 0.001, compared to PBS, ANOVA with repeated measures). Similarly, the plantar inoculation of B16-F10 cells reduces the latency to noxious thermal stimuli (Figure 2B). On the 14th day, the latency drops 15+0.0% in relation to the basal latency at day 0 (p = 0.0453).

### Acute treatment with morphine, recombinant Phα1β, and their combination ameliorate mechanical hyperalgesia induced by B16-F10 inoculation

**Figure 3.**
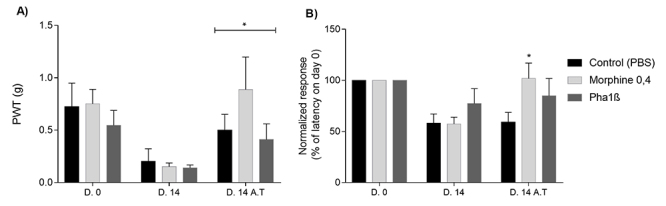
Antinociceptive effect of morphine and recombinant Phα1β on mechanical and thermal hyperalgesia in mice with melanoma. Day 0 (D.0) represents the measurements immediately before B16-F10 cell inoculation. Day 14 (D.14) shows data on the inoculation of B16-F10 cells at the right hind paw, which induces a reduction on both **(A)** paw withdraw threshold (PWT) and in **(B)** the withdrawal latency for a thermal stimulus indicating mechanical and thermal hyperalgesia, respectively. Fourteen days after B16-F10 inoculation (D.14 after treatment - A.T.), the treatment with morphine (0.4 nmol/site, intrathecal) or recombinant Phα1β (30 pmol/site, intrathecal) significantly reverses the mechanical hyperalgesia whereas only morphine was able to reverse thermal hyperalgesia. Data are reported as mean ± S.E.M. *p < 0.05 compared to data on D.14 (ANOVA with repeated measures), data are expressed as mean ± SEM (n = 8-10 animals per group).

To determine the nature of the antinociceptive interaction between morphine and recombinant Phα1β in a cancer pain model, compounds were first administered separately and tested using the PWT and thermal noxious stimulus on rats fourteen days after melanoma induction. PWT and thermal thresholds were assessed 30 minutes after drug treatment. Previous time-response data using similar melanoma-induced pain model suggested that at this time both morphine and Phα1β display consistent antinociceptive effect [15]. Both Phα1β (30 pmol/site, i.t.) and morphine (0.4 nmol/site, i.t.) were efficient on the reversal of melanoma-induced mechanical hyperalgesia (Figure 3A). The paw withdrawal threshold increased from 0.151 ± 0.035 to 0.887 ± 0.310 and from 0.139 ± 0.029 to 0.409 ± 0.151 after morphine and Phα1β treatments, respectively (p = 0.016). At this dosage, however, only morphine was efficient on the reversal of thermal hyperalgesia induced by tumor inoculation (Figure 3B). 

Thus, we decided to explore the dose-response effect of morphine, Phα1β and their combination, given by intrathecal route, on the reversal of melanoma-induced mechanical hyperalgesia. We tested the hypothesis that when given in combination with morphine, the Phα1β toxin causes a leftward shift in the dose-response curve greater than what should be expected if the antinociceptive interaction of both drugs was merely additive. Morphine (7900, 34000 and 130000 pmol/site) dose-dependently increased PWT 30 minutes after its administration (Figure 3A); morphine achieved full efficacy at 130000 pmol/site, i.t. Phα1β (2-100 pmol/site, i.t.) also dose-dependently increased PWT 30 minutes after administration (Figure 3B). Phα1β reaches 60.913 ± 30.918% of MPE at the higher tested dose (100 pmol/site, i.t.). The ED50 values with 95% confidence intervals of morphine and Phα1β were 25 nmol/site (1-562) and 0.031 nmol/site (0.004-0.223) (Table 1). 

**Table 1.** ED_50_ (50% antinociceptive doses) of the agents in melanoma-induced mechanical hypersensitivity.

	ED_50_ (morphine)	ED_50_ (Phα1β)	ED_50_ (Z_add_)	ED_50_ (Z_mix_)
nMol/site	25	0.031	7.4	3.1
95% confidence intervals	(1-562)	(0.004-0.223)	(1.1-44.8)	(0.02-501)

ED_50_ (50% antinociceptive doses) are expressed in nmol/site. The ED_50_ was determined from the dose-response curves. The theoretical additive (Z_add_) was calculated based on the dose-response curves of morphine and Phα1β alone. The combined (Z_mix_) was determined from the experimentally determined dose-response curves of the combination. Values in parenthesis are 95% confidence intervals.

### The antinociceptive interaction between morphine and recombinant Phα1β is synergic

**Figure 4.**
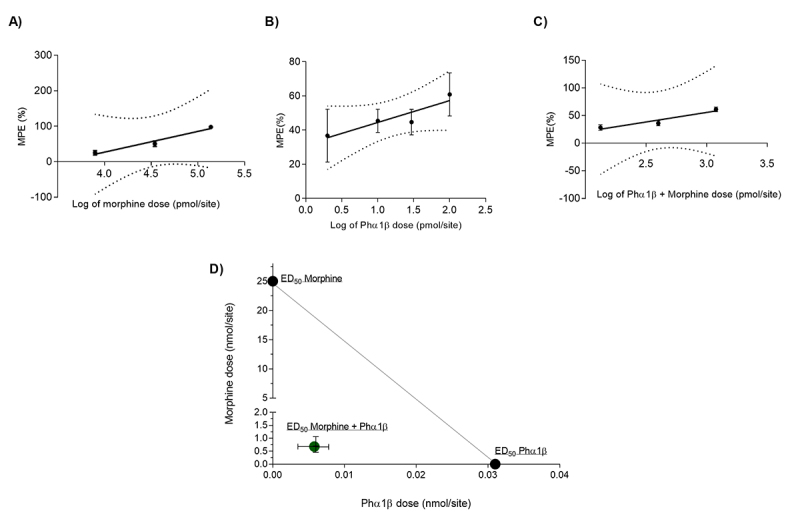
Dose-response analyses of morphine, recombinant Phα1β, and their combination of melanoma-induced mechanical hypersensitivity. **(A)** Linear regression data for increasing concentrations of morphine, **(B)** recombinant Phα1β, **(C)** and their combination in the antinociceptive effect on melanoma-induced mechanical hyperalgesia. Points express the mean ± S.E.M. of the normalized responses given in maximum possible effect (MPE%). Regression lines with their respective 95% confidence intervals are also shown (doted lines). n = 4-7 animals per dose. **(D)** Isobologram for the ED_50_ of morphine (y-axis) plotted against Phα1β (x-axis). The points over the axis denote the ED_50_ of Phα1β and morphine administered alone. The line connecting the ED_50_ values is the theoretical additive line. The point inside the graph denotes the experimentally obtained ED_50_ (with 95% confidence intervals) of combined morphine and Phα1β which indicates a synergistic interaction. Confidence intervals for the theoretical additive and isobol point are shown and can be found in Table 1.

Thereafter, we performed a dose-response curve of the intrathecal administration of morphine concomitantly with intrathecal Phα1β with the doses of drugs in a fixed proportion to investigate the antinociceptive interaction of these two drugs. The proportion of doses of individual agents in the pairs was designed to minimize the variance of the theoretical additive ED50 that is expected if the drugs are used together [19, 22] (see “Methods” section). After estimating the variances of the ED50 values from the effect of drugs given alone, the calculated “f” was 0.29 and the dosage of the two components on each drug pair is presented at Table 2. Figure 3C shows the dose-response-curves of the antinociceptive effect of the associated drugs. The experimentally obtained ED50 value (Zmix) with 95% confidence intervals of morphine combined with Phα1β was 3.1 (0.02-501) nmol/site, (Table 1). The Zmix was significantly lower than the calculated theoretical additive ED50 (Zadd, p < 0.05, student's t-test), which indicates that the combination was synergic on the reversal of mechanical hyperalgesia induced by tumor outgrowth (Table 1). The isobologram for 50% MPE also graphically displays the Zmix and its 95% C.I. values (Figure 4D). This value stands below the theoretical line of additivity.

**Table 2.** Doses used in the dose-response curve for the joint treatment with recombinant Phα1β (i.t.) and morphine (i.t.).

Drug pair	Morphine (pmol/site)	Phα1β (pmol/site)	Composed drug pair (pmol/site)
1	132	1	133
2	396	2.9	398.9
3	1187	8.9	1195.9

### Synergistic attenuation of mechanical hyperalgesia by morphine and Phα1β is not accompanied by severe side effects

**Figure 5.**
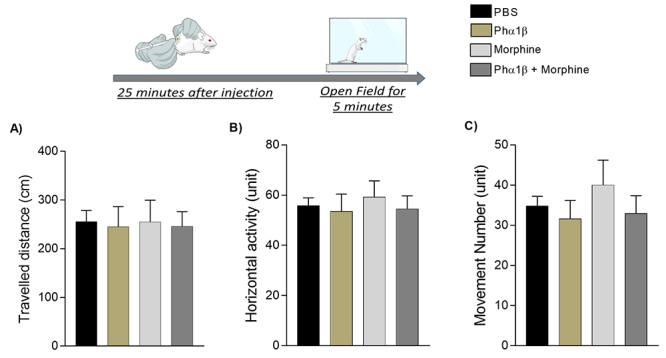
Antinociceptive doses of recombinant Phα1β and morphine (alone or in combination) cause no alterations in spontaneous motor performance in mice. Top image: experimental test scheme. Drugs were injected at doses necessary to cause 50% of MPE in the mechanical hypersensitivity assays. The open-field test was performed 20 minutes after treatment. Data were recorded within 5 minutes. **(A)** Total travelled distance. **(B)** The total number of movements (including flinch, tail-flick, grooming, jumping). **(C)** The total duration of the movements. Data are expressed as mean ± SEM (n = 6 animals per group).

**Figure 6.**
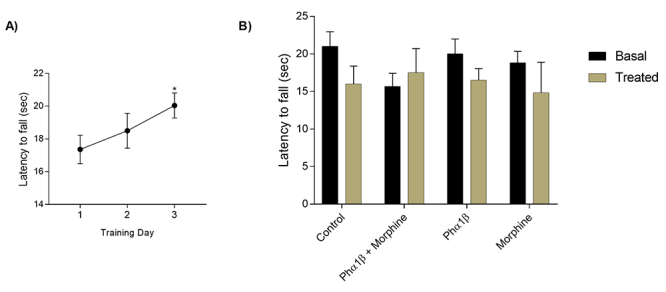
Antinociceptive doses of recombinant Phα1β and morphine (alone or in combination) cause no alterations on forced motor performance in mice. **(A)** For three consecutive days once a day, mice were placed in a rotating cylinder under constant acceleration in the rotarod device. The average latency time to fall increased along the three days (*p = 0.011 compared to the first day) indicating motor learning. **(B)** Thereafter, mice that underwent the protocol shown in A were allocated to receive morphine (25 nmol/site), recombinant Phα1β (0.031 nmol/site), or a combination of the two drugs (3.1 nmol/site), doses required to cause 50% of MPE (Table 1). Measurements were made before (black bars) and 30 minutes after (gray bars) treatment. No statistical difference was observed. Data are expressed as mean ± S.E.M. (n = 6 animals per group).

In light of the synergistic antinociceptive interaction between morphine and Phα1β we next analyzed the effects of both compounds alone or in combination on motor function in naive C57BL/6J mice (without melanoma). The drugs alone or combined were tested at a dose necessary to cause 50% of MPE in the melanoma-induced hyperalgesia assay (Table 1) and the injection was performed 30 minutes before the tests. Morphine and Phα1β, alone or in combination, exhibited no effect on either the spontaneous or in the forced locomotor activity of the animals, as assessed in the open-field (Figure 5A to 5C) and the rota-rod tests (Figure 6), respectively.

## Discussion

This study shows that intrathecally coadministered morphine and recombinant Phα1β interact synergistically to produce analgesia in a model of melanoma-induced pain. The potency of the mixture to ameliorate mechanical hyperalgesia was about twice fold higher than expected if the interaction between morphine and Phα1β was merely additive. Therefore, significantly lower doses of the mixture are required to achieve a specified analgesic effect level. Moreover, the mixture did not elicit significant motor alterations at doses necessary to cause analgesia. Using a similar model of pain in mice, Rigo and co-workers [15] showed that intrathecal injection of 30 pmol/site recombinant toxin and 30 nmol/site morphine significantly reverse melanoma-induced mechanical hyperalgesia for 6 and 1 hour, respectively. When injected in conjunction at a similar route (e.g. intrathecal) and at similar doses morphine and Phα1β showed no additive time extension on their antinociceptive effectiveness [11] suggesting that the synergism observed in our study might not account for an extension in the duration of drug’s action. 

To investigate the antinociceptive interaction between intrathecal morphine and Phα1β, isobolographic analysis was adopted in the present study, which is considered a gold standard to address the magnitude of the interaction between drugs with overtly similar effects, in this case, the antinociceptive effect [21,22]. Using this analysis, the results in these experiments revealed that the experimental ED50 of the mixture is lower than the theoretically additive ED50 (Table 1, and isobologram). This represents a significant degree of synergistic interaction. From a mechanistic standpoint of the two drugs, our findings were comparable to what was observed previously by Omote [23] who showed, also by isobolographic analysis, a similar degree of antinociceptive interaction between ω-CgTx (a specific N-type calcium blocker) and morphine in a model of acute pain [24]. Spinally delivered L- and P/Q-type Ca2+-channel blockers also potentiate morphine analgesia in mice [23,25]. Of note, Phα1β is not a selective blocker of N-type calcium channels but also can blocks L, P/Q and R subtypes of VGCC's [2].

Despite a well-documented number of evidence for the molecular targets of Phα1β and morphine, it has not yet been possible to elucidate the mechanisms to interpret the molecular events underlying the synergistic behavioral effects induced by co-administration of the two drugs because they are the result of the complex interaction and integration of different neural pathways. Although the precise mechanism of synergism was not addressed in this study, the observed synergism reinforces a straightforward observation that Phα1β and morphine act at distinct and separate sites to produce antinociception. Moreover, given that in our study morphine and Phα1β were administered in conjunction at the subarachnoid space, it is unlikely that pharmacokinetic events account for this analgesic cooperative interaction, suggesting that the overlapping point of cooperation of the two drugs occurs downstream to their primary targets.

Clinical data on melanoma show that about 7% of patients experienced pain whereas metastatic melanoma is associated with excruciating pain and more than 50% of these patients require palliative care and morphine treatment [26]. In our experiments, we observed a drop in the mechanical threshold in all animals. Moreover, this increased sensitivity was observed on both inoculated (ipsilateral) and non-inoculated (contralateral) hind-paw indicating a secondary sensitized site than the primary tumor. The efficacy of morphine and Phα1β, when given alone in the reversal of melanoma-induced mechanical hyperalgesia, corroborates previous finding [15] and is in line with the role of µ-opioid receptors and VGCCs in the release of algogenic neurotransmitters from nociceptors into the spinal cord which allows the ascending of nociceptive inputs on cancer pain [27]. Conversely, Phα1β was not able to significantly relieve the melanoma-induced thermal hyperalgesia, which could not be seen for higher doses of Phα1β either (data not shown). This is in agreement with previous observations showing that intrathecally administered calcium channel blockers were poorly effective to alleviate thermal hyperalgesia in acute pain models [23]. Of note, in our study, we observed an expressive variance in the measures for thermal withdrawal latency. We therefore cannot exclude the hypothesis that an antinociceptive potentiation between Phα1β and morphine on thermal hyperalgesia could also exist. However, under these circumstances of low reproducibility, this effect could be underestimated.

The reported side effects of morphine include sedation, respiratory depression, nausea and vomiting, and perhaps most common of all, constipation [6]. It has been previously shown that co-administration of Phα1β with morphine partially restores the reduction of intestinal mobility under repeated morphine treatment [10]. The main Phα1β adverse effects reported so far in rodents include motor disturbances such as tremor, paralysis and tail serpentine movements [28]. Our study expands the repertoire of information on adverse events for combined Phα1β+morphine. No motor deficits were observed for any of the drugs (given at doses that cause 50% of maximum antinociceptive effect) even if they are combined most likely reflecting the dose reduction of both compounds necessary to cause analgesia.

Our study presents limitations. We did not explore long-term features of continuous treatment with the mixture. The orthotropic tumor inoculation used in our study is a fast-growing tumor that becomes lethal to mice in a few days, thus future studies using long-lasting chronic models are necessary to address this question. Adverse effects were tested on naive animals rather than in B16-F10 inoculated animals. This choice was due to the interference that melanoma *per se* could do on mice locomotion becoming, thus, a confounding factor for motor deficits associated with drug treatments. To reduce the number of used animals to a minimum (following the 3R's recommendations), only a single dose of each drug alone or combined was accessed for investigation of adverse events. Albeit we could not obtain an interaction index for adverse events - such as for antihyperalgesic effect - we were able to indirectly infer for a lack of potentiating of drugs when they are used together at an equieffective analgesic dose of the compounds. Future preclinical studies are also necessary to elucidate the hemodynamic effects of Phα1β given alone or in conjunction with morphine. Investigation of this set of adverse events is mandatory before this molecule can be translated into human beings. 

## Conclusion

In conclusion, Phα1β recombinant toxin administrated as an adjuvant to morphine significantly and safely potentiates analgesia in a model of cancer-related pain as a consequence of a synergistic analgesic interaction. These findings expand the repertoire of analgesic options and strengthen the strategy of combining drugs that act on different targets to control pain.

## References

[B1] Lauria PSS, Villarreal CF, Casais-e-Silva LL (2020). Pain modulatory properties of Phoneutria nigriventer crude venom and derived peptides: a double-edged sword. Toxicon.

[B2] Vieira LB, Kushmerick C, Hildebrand ME, Garcia E, Stea A, Cordeiro MN, Richardson M, Gomez MV, Snutch TP (2005). Inhibition of high voltage-activated calcium channels by spider toxin PnTx3-6. J Pharmacol Exp Ther.

[B3] Tonello R, Fusi C, Materazzi S, Marone IM, De Logu F, Benemei S, Gonçalves MC, Coppi E, Castro-Junior CJ, Gomez MV, Geppetti P, Ferreira J, Nassini R (2017). The peptide Phα1β, from spider venom, acts as a TRPA1 channel antagonist with antinociceptive effects in mice. Br J Pharmacol.

[B4] Rigo FK, Rossato MF, Borges V, da Silva JF, Pereira EMR, de Ávila RAM, Trevisan G, Dos Santos DC, Diniz DM, Silva MAR, de Castro CJ, Cunha TM, Ferreira J, Gomez MV (2020). Analgesic and side effects of intravenous recombinant Phα1β. J Venom Anim Toxins incl Trop Dis.

[B5] Magee David, Bachtold S, Brown M, Farquhar-Smith P (2019). Cancer pain: where are we now?. Pain Manag.

[B6] Plante GE, VanItallie TB (2010). Opioids for cancer pain: the challenge of optimizing treatment. Metabolism.

[B7] Gilron I, Jensen TS, Dickenson AH (2013). Combination pharmacotherapy for management of chronic pain: from bench to bedside. Lancet Neurol.

[B8] Tajerian M, Millecamps M, Stone LS (2012). Morphine and clonidine synergize to ameliorate low back pain in mice. Pain Res Treat.

[B9] Alicino I, Giglio M, Manca F, Bruno F, Puntillo F (2012). Intrathecal combination of ziconotide and morphine for refractory cancer pain: a rapidly acting and effective choice. Pain.

[B10] Tonello R, Rigo F, Gewehr C, Trevisan G, Pereira REM, Gomez MV, Ferreira J (2014). Action of Phα1β, a peptide from the venom of the spider Phoneutria nigriventer, on analgesic and adverse effects caused by morphine in mice. J Pain.

[B11] Tonello R, Trevisan G, Luckemeyer D, Castro-Junior CJ, Gomez MV, Ferreira J (2020). Phα1β, a dual blocker of TRPA1 and Cav2.2, as an adjuvant drug in opioid therapy for postoperative pain. Toxicon.

[B12] Rigo FK, Dalmolin GD, Trevisan G, Tonello R, Silva MA, Rossato MF, Klafke JZ, Cordeiro MN, de Castro CJ, Montijo D, Gomez MV, Ferreira J (2013). Effect of ω-conotoxin MVIIA and Phα1β on paclitaxel-induced acute and chronic pain. Pharmacol Biochem Behav.

[B13] Zimmermann M (1983). Ethical guidelines for investigations of experimental pain in conscious animals. Pain.

[B14] Mestre C, Pélissier T, Fialip J, Wilcox G, Eschalier A (1994). A method to perform direct transcutaneous intrathecal injection in rats. J Pharmacol Toxicol Methods.

[B15] Rigo FK, Trevisan G, Rosa F, Dalmolin GD, Otuki MF, Cueto AP, de Castro CJ, Romano-Silva MA, Cordeiro MN, Richardson M, Ferreira J, Gomez MV (2013). Spider peptide Ph a 1 b induces analgesic effect in a model of cancer pain. Cancer Sci.

[B16] Hargreaves K, Dubner R, Brown F, Flores C, Joris J (1988). A new and sensitive method for measuring thermal nociception in cutaneous hyperalgesia. Pain.

[B17] Chaplan SR, Bach FW, Pogrel JW, Chung JM, Yaksh TL (1994). Quantitative assessment of tactile allodynia in the rat paw. J Neurosci Methods.

[B18] Dixon WJ (1980). Efficient analysis of experimental observations. Annu Rev Pharmacol Toxicol.

[B19] Tallarida RJ (2000). Drug synergism and dose-effect data analysis.

[B20] Tallarida RJ (2001). Drug synergism: its detection and applications. J Pharmacol Exp Ther.

[B21] Tallarida RJ (2006). An overview of drug combination analysis with isobolograms. J Pharmacol Exp Ther.

[B22] Foucquier J, Guedj M (2015). Analysis of drug combinations: current methodological landscape. Pharmacol Res Perspect.

[B23] Fukuizumi T, Ohkubo T, Kitamura K (2003). Spinally delivered N-, P/Q- and L-type Ca2+-channel blockers potentiate morphine analgesia in mice. Life Sci.

[B24] Omote K, Kawamata M, Satoh O, Iwasaki H, Namiki A (1996). Spinal antinociceptive action of an N-type voltage-dependent calcium channel blocker and the synergistic interaction with morphine. Anesthesiology.

[B25] Omote K, Sonoda H, Kawamata M, Iwasaki H, Namiki A (1993). Potentiation of antinociceptive effects of morphine by calcium-channel blockers at the level of the spinal cord. Anesthesiology.

[B26] Negin BP, Riedel E, Oliveria SA, Berwick M, Coit DG, Brady MS (2003). Symptoms and signs of primary melanoma: important indicators of Breslow depth. Cancer.

[B27] Khasabova IA, Stucky CL, Harding-Rose C, Eikmeier L, Beitz AJ, Coicou LG, Hanson AE, Simone DA, Seybold VS (2007). Chemical interactions between fibrosarcoma cancer cells and sensory neurons contribute to cancer pain. J Neurosci.

[B28] Deer TR, Pope JE, Hanes MC, McDowell GC (2019). Intrathecal therapy for chronic pain: a review of morphine and ziconotide as firstline options. Pain Med.

